# The Impact of Beauty Perception on Sexual Quality of Life in Women with Stoma: A Cross-Sectional Study

**DOI:** 10.1007/s10508-025-03407-9

**Published:** 2026-03-23

**Authors:** Nazife Gamze Özer Özlü, Fatma Vural, Deniz Cenan

**Affiliations:** 1 Faculty of Nursing, Department of Surgical Nursing, Dokuz Eylül University, 35340 Balçova/İnciraltı, Izmir, Turkey; 2 Department of General Surgery, Dokuz Eylül University Hospital, Balçova/İnciraltı, Izmir, Turkey

**Keywords:** Stoma, Body image, Sexual quality of life, Beauty perception, Psychosocial support

## Abstract

Women with a stoma may experience negative effects on their sexual quality of life and well-being due to significant changes in body image and perceptions of beauty. This study aimed to investigate the effect of beauty perception on the sexual quality of life of female stoma patients. This descriptive, cross-sectional, and predictive study was conducted between December 2023 and December 2024 in the general surgery department of a tertiary hospital. The sample consisted of 82 women with a stoma selected using purposive sampling and data were collected through telephone interviews. Data collection tools included the Beauty Subjective Numerical Assessment, Body Type (Somatotype), Body Image Scale, and Sexual Quality of Life Scale–Women. The findings showed that women’s perceptions of beauty decreased after surgery. Women who preferred mesomorphic body types reported higher body image levels compared to those who preferred endomorphic and ectomorphic body types. However, body type preferences did not have a significant effect on sexual quality of life. Postoperative beauty perception was found to be associated with both body image and sexual quality of life. Furthermore, body image emerged as an important predictor of sexual quality of life. In conclusion, stoma surgery negatively affects women’s perceptions of beauty and contributes to declines in body image and sexual quality of life. Psychosocial support and interventions aimed at improving body image should be integrated into postoperative care.

## Introduction

The word stoma is derived from the Greek, meaning “mouth.” It is defined medically as communication, natural or artificial, between a body cavity and the external environment. Surgical procedures in which stomas are created are given the suffix “ostomy.” Artificial stomas are most formed from the gastrointestinal or urinary tract. Intestinal stomas, such as ileostomies and colostomies, as well as urinary stomas, such as urostomies, represent the most common types of surgically created stomas (Pine & Stevenson, 2014).

A stoma can radically alter a person’s body image, often leading to feelings of inadequacy, loss of self-identity, and diminished bodily trust. Patients frequently report uncertainty and vulnerability in daily life following stoma surgery (Petersén & Carlsson, 2021; Song et al., 2021). Consistent evidence indicates that living with a stoma negatively affects quality of life, particularly in relation to sexuality, with body image disturbances and lifestyle changes playing a central role (Heydari et al., 2023; Lin et al., 2023; Medina-Rico et al., 2019).

For women with colorectal cancer, the presence of a stoma may be especially challenging. Physical discomfort, concerns related to intimacy, and perceived devaluation of sexuality are commonly reported after surgery. Odors, sounds, and altered bodily functions can complicate acceptance of the stoma and interfere with sexual expression (Tan et al., 2024). Partner responses and acceptance further shape women’s self-confidence and sexual well-being, as fear of rejection may exacerbate body image concerns and feelings of unattractiveness (Tripaldi, 2019).

Research on women’s sexual function and body image following cancer treatment has documented a range of physical and sensory changes, including fatigue, vaginal dryness, decreased libido, and difficulty achieving orgasm. Women may also experience numbness in previously erogenous zones, pain during intercourse, and an overall reduction in sexual pleasure (Che et al., 2022; Sperring et al., 2025). These experiences contribute to emotional distress and negative body-related self-evaluations, reflected in reduced self-perceived attractiveness, diminished confidence in female identity, and altered perceptions of womanhood. Consequently, many women report less frequent and more restricted sexual activity, accompanied by a loss of spontaneity, as surgery is experienced as a profound disruption to the body (Che et al., 2022; Sperring et al., 2025).

Within this context, women’s experiences of sexuality and body image are closely intertwined with broader societal beauty ideals. Beauty standards are shaped through an interaction of biological and sociocultural factors, with gender norms often portraying women as delicate and aesthetically pleasing, reinforcing expectations to conform to specific ideals of appearance (Eisend, 2019; Kurtdaş, 2021). Physical appearance, particularly facial and bodily symmetry, occupies a central role in beauty perception (Harrar et al., 2018; Maymone et al., 2019). When women perceive themselves as failing to meet these ideals, they may encounter social stigma, relational difficulties, and diminished sexual quality of life (Kukkonen et al., 2023). Although sexuality generally enhances overall quality of life, it is especially vulnerable to psychological and emotional disruptions (Grinde, 2022).

In this framework, beauty perception can be understood as an individual’s subjective evaluation of their physical appearance, encompassing self-perceived attractiveness, satisfaction with bodily features, and perceived alignment with personal and sociocultural beauty standards. This multidimensional construct is closely related to, yet distinct from, general body image and is influenced by cultural norms, personal experiences, and significant life events. For women in particular, beauty perception is strongly linked to self-esteem, gender identity, and sexuality, making it a critical component of psychosocial well-being (Castro et al. 2017; Dimitrov et al., 2023; Merino et al., 2024; Rodgers et al., 2023).

Permanent bodily changes such as stoma formation may therefore profoundly challenge beauty perception. Visible alterations to the body and the ongoing presence of a stoma can disrupt previously internalized beauty ideals, leading to negative self-evaluations and reduced body satisfaction. Studies have shown that women living with a stoma frequently report impaired sexual quality of life and unsatisfactory sexual experiences after surgery (Dames et al., 2021; Dou et al., 2023; Kaziga et al. 2021; Ramirez et al., 2014; Sütsünbuloğlu & Vural, 2018; Vural et al., 2016). Nevertheless, clinical care often prioritizes physical recovery and stoma management, while sexuality-related concerns and psychosocial adjustment receive comparatively limited attention (García-Rodríguez et al., 2021; Sütsünbüloğlu & Vural, 2018).

Given that female beauty has historically been closely intertwined with identity and self-worth, disruptions in beauty perception following stoma surgery may have far-reaching implications for women’s sexual lives. However, despite its potential importance, the role of beauty perception in shaping sexual quality of life among women with a stoma remains insufficiently explored (Jayarajah & Samarasekera, 2017).

The current study aimed to examine the relationship between beauty perception and sexual quality of life in women living with a stoma. Specifically, this study sought to investigate (1) changes in women’s perception of beauty following stoma surgery, (2) the associations between beauty perception, body image, and sexual quality of life, and (3) the extent to which beauty perception and body image predict sexual quality of life. It was hypothesized that a more negative perception of beauty would be associated with poorer body image and lower sexual quality of life, and that body image would significantly predict sexual quality of life. By addressing these research questions, the study aims to contribute to a more comprehensive understanding of the psychosocial factors influencing sexual well-being in women with a stoma.

The research questions were as follows:

1. What is the level of beauty perception of women with stoma?
2. Is there a difference between the perception of beauty of women with stoma before and after stoma surgery?
3. How does the perception of beauty affect the sexual quality of life of women with stoma?

We conducted a descriptive, cross-sectional, and predictive study. STROBE checklist: Cross-Sectional studies were conducted, considering the checklist (Uhling et al., 2007).

## Method

### Participants

The study was conducted between December 1, 2023, and December 30, 2024. Female patients who underwent stoma surgery at the tertiary hospital, department of general surgery and were followed up in the stoma therapy unit after surgery were included in the study. This study is based on women’s subjective evaluation to investigate the effect of beauty perception on the sexual quality of life of women with stoma.

The study included adult cisgender women who had a stoma for at least two months, were able to read, speak, and write Turkish, and reported having an active sexual life after stoma surgery with a male partner. Sexual activity in the context of this study was evaluated within the framework of heterosexual (woman–man) relationships.

An active sexual life was operationally defined as engagement in consensual sexual intercourse with a male partner at least once within the previous three months following surgery. Women who reported no sexual activity either before or after stoma creation were excluded from the study.

The sample size calculation was based on the number of patients attending the stoma therapy unit. Approximately 500 individuals with a stoma are followed in this unit annually. The sample size was calculated using G-Power 3.1.9.2 with a 95% confidence interval. The sample size was 82, with a two-way medium effect size. After data were collected from 82 patients, the power analysis was performed again using the study’s data. In the post hoc power analysis performed according to the body image comparison of body type, the two-sided effect size was 3.21, and the power was 1.000.

### Measures and Procedure

The first researcher collected all data through face-to-face telephone interviews. For the telephone interviews, the third researcher, a WOC nurse, called the patients and provided them with preliminary information. The first researcher then contacted the patients and made an appointment for the telephone interview. The telephone interviews were conducted at the appointment time given by the patients to the first researcher. The informed consent form was sent to the patients via WhatsApp prior to the interview, and the patients signed and returned it to the researcher. Since all data was not collected face-to-face, the study was conducted in accordance with the Personal Data Protection Law No. 6698 of the country where the study was conducted. Therefore, the name and photograph of each participant were anonymized.

Data were collected using the “Patient Descriptive Characteristics Form,” “Beauty Subjective Numerical Assessment,” “Body Type (Somatotype),” and “Body Image Scale” to determine women’s perception of beauty and the “Sexual Quality of Life Scale-Woman” to assess women’s sexual quality of life.

*Patient Descriptive Characteristics Form*: This form consists of 11 questions about individuals’ descriptive characteristics, which the researchers developed using literature (Petersén & Carlsson, 2021; Sutsunbuloglu & Vural, 2018; Tan et al., 2024; Tripaldi, 2019). The questions include gender, age, educational status, occupation, marital status, type of stoma, duration of stoma, and reason for stoma surgery.

*Beauty Subjective Numerical Assessment*: The authors developed it from the literature and gave a 0–10-point rating of how the person perceives themself as physically and facially beautiful before and after surgery. Zero indicates she is not considered attractive, and 10 suggests she is very beautiful.

*Body Type (Somatotype)*: The concept of somatotype was introduced by Sheldon (1950), who described three basic body types: endomorphic, mesomorphic, and ectomorphic. In the present study, somatotype was not assessed using anthropometric measurements or clinical classification. Instead, body type preference was evaluated based on women’s subjective perceptions of beauty. During the telephone interview, women were shown standardized visual illustrations representing the three classical somatotypes, accompanied by brief descriptions to ensure comprehension. Women were asked to indicate which body type they perceived as the most aesthetically appealing. This approach aimed to capture women’s aesthetic body type preferences rather than their own physical body composition. Based on their responses, women were categorized into three groups according to their preferred somatotype: endomorphic, mesomorphic, or ectomorphic.

*Body Image Scale:* The 10-item scale was developed to provide a brief and comprehensive assessment of emotional (e.g., self-consciousness), behavioral (e.g., difficulty looking at naked body), and cognitive (e.g., satisfaction with appearance) aspects of body image in cancer patients. It is designed to be used across all cancer types and treatment settings (Hopwood et al., 2001). It briefly and comprehensively assesses emotional (e.g., self-consciousness), behavioral (e.g., difficulty looking at the naked body), and cognitive (e.g., satisfaction with appearance) components of body perception. It is a 4-point scale (0 = not at all and 3 = very much), and the final score is the sum of the scores for the 10 items. A lower score on a scale of 0 to 30 represents a better body image. Turkish reliability and validity study was conducted by Karayurt et al. (2015).

*Sexual Quality of Life Questionnaire–Female (SQOL-F):* It was developed by Symonds et al. (2005) to assess sexual well-being in women. The Turkish reliability and validity study was conducted by Tuğut and Gölbaşı (2010). The scale consists of 18 items rated on a Likert-type scale, with higher scores indicating better sexual quality of life. Items are answered based on sexual experiences during the previous four weeks. Scoring procedures were applied in accordance with the original scale and its validated Turkish version.

### Statistical Analysis

Statistical analyses were performed using IBM SPSS Statistics version 29.0. The distribution of continuous variables was assessed using Kolmogorov–Smirnov and Shapiro–Wilk tests, along with visual inspection of histograms and Q–Q plots. Continuous variables were summarized using means and standard deviations, while categorical variables were presented as frequencies and percentages. Differences between preoperative and postoperative beauty perception scores were examined using the Wilcoxon signed-rank test. Postoperative body image and sexual quality of life scores were compared across body type preference groups using one-way analysis of variance (ANOVA). Given the unequal group sizes, these analyses were interpreted as exploratory. When overall group differences were detected, Tukey post hoc test was used following ANOVA. Associations between beauty perception, body image, and sexual quality of life were evaluated using Pearson correlation coefficients. Multiple linear regression analysis was conducted to examine predictors of postoperative sexual quality of life. Due to the cross-sectional design, regression analyses were interpreted as predictive associations rather than causal effects. Statistical significance was set at *p* < 0.05.

## Results

### Patient Descriptive Characteristics and Women’s Perception of Beauty

The participants were middle-aged women, most of whom were married, housewives, and had a temporary stoma. Colostomy was the most common type of stoma, and cancer was the leading indication for surgery. More than half of the women reported experiencing sexual difficulties following stoma surgery. Before surgery, women reported high perceptions of both physical and facial beauty; however, these perceptions markedly decreased in the postoperative period. Postoperative body image scores indicated a generally impaired body image following stoma surgery (Table 1).

**Table 1:** Patient descriptive characteristics and women’s perception of beauty

Characteristics	n	%	M ± SD	Min	Max
*Age* (in years)	82	100	51.70 ± 10.71	27.00	69.00
*Education level*					
Literate	7	8.5			
Primary Education	34	41.5			
Secondary Education	28	34.1			
Higher Education	13	15.9			
*Occupation*					
Housewife	54	65.9			
Worker	6	7.3			
Officer	5	6.1			
Self-employed	2	2.4			
Retired	15	18.3			
*Marital status*					
Married	67	81.7			
Single	15	18.3			
*Kind of stomas*					
Temporary	59	72.0			
Permanent	23	28.0			
*Type of stoma*					
Ileostomy	17	20.7			
Colostomy	60	73.2			
Urostomy	5	6.1			
*Indication for stoma surgery*					
Cancer	48	58.5			
Inflammatory bowel diseases	16	19.5			
Trauma	8	9.8			
Other (Infections, vascular conditions etc.)	10	12.2			
*Sexual difficulties after stoma surgery*					
Yes	42	51.2			
No	40	48.8			
*Duration of stoma* (in months)	82	100.00	11.93 ± 19.93	3.00	168.00
*Body type (Somatotype)*					
Endomorph	16	19.5			
Mesomorph	52	63.4			
Ectomorph	14	17.1			
*Preoperative perception of physical beauty* (0–10)	82	100.0	8.93 ± 1.59	3.00	10.00
*Preoperative perception of facial beauty* (0–10)	82	100.0	8.59 ± 2.08	0.00	10.00
*Postoperative perception of physical beauty* (0–10)	82	100.0	4.51 ± 2.81	0.00	10.00
*Postoperative perception of facial beauty* (0–10)	82	100.0	5.84 ± 3.40	0.00	10.00
*Postoperative body image* (0–10)	82	100.0	29.89 ± 9.45	10.00	50.00

### Changes in Women’s Perception of Beauty Before and After Surgery

Consistent with the study hypothesis, women’s perceptions of both physical and facial beauty significantly declined after stoma surgery. These findings indicate that stoma surgery is associated with a substantial deterioration in women’s subjective beauty perception (Table 2).

**Table 2:** Comparison of women’s perception of beauty before and after surgery

	Preoperative Period	Postoperative Period	Test statistics	
	M ± SD	M ± SD	Z	*p* value
Perception of physical beauty	8.93 ± 1.59	4.51 ± 2.81	− 7.47	< 0.001
Perception of facial beauty	8.59 ± 2.08	5.84 ± 3.40	− 5.49	< 0.001

Z = Wilcoxon Signed Rank Test

### Postoperative Body Image and Sexual Quality of Life According to Preferred Body Type

Postoperative body image scores differed significantly according to the body type perceived as most aesthetically attractive (Table 3). Post hoc Tukey analysis showed that this significant group difference was driven by the comparison between the mesomorphic and endomorphic preference groups, with higher postoperative body image scores observed in the mesomorphic group (p = 0.007). No significant differences were found in comparisons involving the ectomorphic group. Due to unequal group sizes, these findings are interpreted as exploratory. In contrast, sexual quality of life scores did not differ significantly across preferred body type groups.

**Table 3:** Comparison of postoperative body image and sexual quality of life by body type

Body type (Somatotype)	Body Image		Quality of sexual life	
	M ± SD	Test statistics *p value*	M ± SD	Test statistics *p* value
Endomorph (I)	24.50 ± 8.67	F = 5.24	49.58 ± 22.59	F = 2.776
Mesomorph (II)	32.25 ± 8.85	p = 0.007	62.45 ± 22.51	*p* = 0.068
Ectomorph (III)	27.28 ± 9.87	I < II	50.71 ± 24.61	

### Associations Between Beauty Perception, Body Image, and Sexual Quality of Life

Preoperative perceptions of physical and facial beauty were not associated with postoperative body image or sexual quality of life. In contrast, postoperative perceptions of physical and facial beauty were strongly associated with both body image and sexual quality of life. Lower postoperative beauty perception was related to poorer body image and reduced sexual quality of life, supporting the hypothesized relationship between postoperative self-perception and psychosocial outcomes (Table 4).

**Table 4:** Correlation of the perception of beauty with the postoperative body image and the sexual quality of life

Perioperative Period	Perception of Beauty	Body Image		Sexual Quality of Life	
		*r*	*p*	*r*	*p*
Preoperative	Perception of physical beauty	− 0192	0.085	0.04	0.179
	Perception of facial beauty	− 0.123	0.269	0.04	0.683
Postoperative	Perception of physical beauty	− 0.579	< 0.001	-0.47	< 0.001
	Perception of facial beauty	− 0.602	< 0.001	− 0.34	0.002

### Predictors of Postoperative Body Image and Sexual Quality of Life

Regression analyses demonstrated that body image was a key predictor of sexual quality of life in the postoperative period. Perceptions of physical and facial beauty and body type did not independently predict body image. The overall regression model explained a substantial proportion of the variance in sexual quality of life, supporting the central role of body image in postoperative sexual well-being (Table 5).

**Table 5:** Multiple linear regression analysis predicting sexual quality of life

	B	Standard error	β	*t*	*p*
*Constant*	22.03	14.64	-	1.50	0.136
Perception of physical beauty	− 1.73	0.94	− 0.20	− 1.83	0.071
Perception of facial beauty	0.76	0.80	0.11	0.95	0342
Body type (Somatotype)	− 1.23	3.41	− 0.03	− 0.36	0.718
Body image	1.39	0.29	0.56	0.56	< 0.001
R	0.63				
R^2^	0.40				
*F*	13.13				
*p*	< 0.001				
DW (1.5–2.5)	1.79				

## Discussion

This study examined the associations between pre- and postoperative beauty perceptions, body image, and sexual quality of life among women with a stoma. Overall, the findings indicate that stoma surgery is accompanied by substantial changes in women’s perceptions of beauty, body image, and sexual well-being. In the postoperative period, lower beauty perceptions were associated with poorer body image and reduced sexual quality of life. In addition, body type preference was related to postoperative body image, although no significant association was observed with sexual quality of life.

Women’s perceptions of beauty declined markedly following stoma surgery, highlighting the psychological impact of bodily changes associated with stoma formation. Lower postoperative beauty perception was associated with poorer body image, suggesting increased dissatisfaction with physical appearance after surgery. These findings are consistent with previous longitudinal research demonstrating that individuals with ostomies report lower body image scores and higher levels of psychological distress compared with those without ostomies (Song et al., 2021). Similarly, qualitative studies have shown that female cancer patients often experience discomfort with their bodies and challenges related to femininity due to treatment-related physical changes (Kocan et al., 2023). Together, these findings underscore the close relationship between altered beauty perception and psychological well-being and highlight the importance of addressing psychosocial needs in postoperative care.

Body image and sexual quality of life, while related, represent distinct dimensions influenced by multiple individual and psychosocial factors. In the present study, women who perceived a mesomorphic body type as the most aesthetically attractive reported more positive postoperative body image compared with those who preferred endomorphic or ectomorphic body types. This finding suggests that postoperative body image may be shaped by the degree of alignment between bodily changes following surgery and internalized aesthetic ideals. In contrast, preferred body type was not significantly associated with sexual quality of life. This result aligns with previous literature indicating that sexual well-being is influenced by a complex interplay of factors beyond body image alone, including relationship quality, partner support, emotional intimacy, and sociocultural norms (Nowosielski et al., 2019). Accordingly, body type preference may be relevant for body image perception but appears to have a more indirect role in sexual quality of life.

The study also demonstrated that preoperative perceptions of physical and facial beauty were not significantly associated with postoperative body image or sexual quality of life. In contrast, postoperative beauty perception showed moderate to strong associations with both outcomes. Approximately half of the women reported sexual difficulties after stoma surgery, highlighting the multifaceted challenges faced in the postoperative period. These findings are consistent with prior studies documenting sexual dissatisfaction and dysfunction among individuals living with a stoma (Sütsünbüloğlu & Vural, 2018). Previous research suggests that sexual experiences after stoma surgery are influenced not only by physical factors but also by psychological processes and partner responses (Ramirez et al., 2014; Tripaldi, 2019). Partner acceptance, emotional closeness, and perceived support have been identified as key contributors to sexual adjustment following stoma surgery (García-Rodríguez et al., 2021; Vural et al., 2016).

Finally, regression analysis demonstrated that body image was a key factor associated with sexual quality of life, with women reporting more positive body image also reporting better sexual well-being. This finding reinforces the central role of body-related self-perceptions in women’s sexual adjustment after stoma surgery and is consistent with previous studies across different clinical populations showing strong links between body image and sexual satisfaction (Dames et al., 2021; Miguel et al., 2025). Importantly, these results suggest that sexual quality of life following stoma surgery may be less dependent on aesthetic ideals per se and more strongly influenced by how women perceive, accept, and emotionally relate to their altered bodies. Although the cross-sectional design precludes causal conclusions, the findings highlight body image as a potentially modifiable target for psychosocial and multidisciplinary interventions aimed at improving sexual well-being in women living with a stoma.

### Strengths, Limitations, and Future Directions

Several limitations of this study should be acknowledged. First, the cross-sectional design does not allow causal inferences regarding the relationships between beauty perception, body image, and sexual quality of life; the observed associations reflect a single time point, and longitudinal studies are needed to clarify temporal and bidirectional effects.

Second, the sample consisted exclusively of cisgender women in heterosexual relationships. While this increased sample homogeneity, it limits the generalizability of the findings to women with different sexual orientations, relationship structures, or gender identities.

Third, analyses involving preferred body type were constrained by unequal and relatively small group sizes, particularly in the endomorphic and ectomorphic preference groups. Although parametric assumptions were met, these imbalances may have reduced statistical power; therefore, findings related to body type preference should be interpreted as exploratory.

In addition, the single-center design and specific cultural context may further limit generalizability, as perceptions of beauty, body image, and sexuality are culturally influenced. Finally, all measures relied on self-report, which may be subject to social desirability and recall bias. Future studies using larger, more diverse samples and mixed-method or longitudinal designs are warranted.

### Conclusion

This study indicates that women with a stoma report lower perceptions of beauty in the postoperative period and that these perceptions are associated with body image and sexual quality of life. A strong association was observed between body image and sexual quality of life, with more positive body image related to better sexual well-being. In contrast, preferred body type was associated with postoperative body image but was not significantly related to sexual quality of life.

These findings underscore the clinical relevance of body image and beauty perception in the postoperative adjustment of women with a stoma. Addressing body image concerns through psychosocial and multidisciplinary interventions may contribute to improved sexual quality of life and overall well-being. Importantly, the observed associations should not be interpreted as causal due to the cross-sectional design of the study.

Future longitudinal and interventional research is needed to clarify the temporal relationships among beauty perception, body image, and sexual quality of life, and to evaluate the effectiveness of targeted psychosocial and body image–focused interventions in women living with a stoma.
